# GAN-Based Novel Approach for Generating Synthetic Medical Tabular Data

**DOI:** 10.3390/bioengineering11121288

**Published:** 2024-12-18

**Authors:** Rashid Nasimov, Nigorakhon Nasimova, Sanjar Mirzakhalilov, Gul Tokdemir, Mohammad Rizwan, Akmalbek Abdusalomov, Young-Im Cho

**Affiliations:** 1 Artificial Intelligence, Tashkent State University of Economics, Tashkent 100066, Uzbekistan; rashid.nasimov@tsue.uz; 2 Department of Software Information Technologies, Tashkent University of Information Technologies Named After Muhammad Al-Khwarizmi, Tashkent 100200, Uzbekistan; nigorakhon.nasimova@tuit.uz (N.N.); mirzaxalilov86@tuit.uz (S.M.); 3 Department of Computer Engineering, Faculty of Engineering, Cankaya University, 06790 Ankara, Turkey; gtokdemir@cankaya.edu.tr; 4 Centre of Excellence for Electric Vehicle and Related Technologies, Department of Electrical Engineering, Delhi Technological University, Delhi 110042, India; rizwan@dce.ac.in; 5 Department of Computer Engineering, Gachon University, Sujeong-gu, Seongnam-si 461-701, Gyeonggi-do, Republic of Korea

**Keywords:** GAN, synthetic medical tabular data, statistical data, custom loss function

## Abstract

The generation of synthetic medical data has become a focal point for researchers, driven by the increasing demand for privacy-preserving solutions. While existing generative methods heavily rely on real datasets for training, access to such data is often restricted. In contrast, statistical information about these datasets is more readily available, yet current methods struggle to generate tabular data solely from statistical inputs. This study addresses the gaps by introducing a novel approach that converts statistical data into tabular datasets using a modified Generative Adversarial Network (GAN) architecture. A custom loss function was incorporated into the training process to enhance the quality of the generated data. The proposed method is evaluated using fidelity and utility metrics, achieving “Good” similarity and “Excellent” utility scores. While the generated data may not fully replace real databases, it demonstrates satisfactory performance for training machine-learning algorithms. This work provides a promising solution for synthetic data generation when real datasets are inaccessible, with potential applications in medical data privacy and beyond.

## 1. Introduction

In recent years, the generation of synthetic data has emerged as a prominent trend in the medical domain. This is because accessing real medical databases is very challenging, simply because medical records include private or sensitive data with special protections from the Health Insurance Portability and Accountability Act (HIPAA) [1] and General Data Protection Regulation (GDPR) [2]. In developed countries, hospitals have adopted Electronic Health Records (EHRs) to store patients’ medical histories, reduce the need for physical records and manual paperwork, streamline processes like billing, scheduling and lab results and give the opportunity to medical professionals to easily access and share patient information across different healthcare facilities. Considering that the majority of hospital systems are integrated, every developed country currently possesses a massive database containing hundreds of thousands, if not millions, of patient records. Nevertheless, due to privacy policy, this database is not publicly available. Traditional methods of data anonymization, such as data masking, pose a serious threat to data utility while also risking privacy [3]. To solve this problem, synthetic data is the best choice, which can help to anonymize real hospital data and protect sensitive information while maintaining the statistical features of actual data. Despite its several disadvantages such as limited realism—synthetic data may fail to capture the true variability and complex patterns of real-world medical data, reduced generalizability—synthetic data might not include rare or unforeseen conditions present in real-world datasets [4]; synthetic data can be an important factor for conducting research, especially in the field of healthcare since synthetic data give some benefits such as improving data accessibility and high-quality data augmentation. For this reason, in the last several years, the number of research papers in the field of generating synthetic medical data has increased significantly, and would be more useful in the future. However, real databases are used as the primary tool in all practical methods, including statistical and probabilistic methods that employ real databases to obtain the necessary distributions. However, as previously mentioned, an unauthorized individual cannot obtain access, even if their purpose is to convert real databases into a synthetic one. Such access is only available to individuals who have obtained special permission under special agreements. Because of this, extremely few studies use a database that is not openly accessible to generate synthetic data [5,6,7]. Almost all other studies use open databases. The question arises what is the purpose of creating a synthetic database from an already open database. There are two goals: the first is to augment the existing small database, and the second is to prove the effectiveness of the proposed method.

However, no matter how effective the proposed method is, it cannot create any synthetic data without the author having direct access to the real database. Therefore, despite the huge amount of research on synthetic data generation in recent years, very few databases are being made available for open use. That is, the problem of data shortage here is still an urgent problem to solve. As a solution to this problem, the use of statistical information about real tabular databases, rather than the database itself, is proposed to create synthetic data. This is because today there is statistical information about a huge number of important databases that are not publicly available. In particular, if there are databases collected within the framework of a specific project or research, then full statistical information about them is presented in the articles. For example, in [8,9,10,11], a lot of statistical information is given about a very large database while the database is not openly available. This study introduces a novel method for generating fully synthetic databases using only statistical data, leveraging modified GANs. To the best of our knowledge, this approach represents the first attempt to generate synthetic tabular data directly from statistical data using deep learning models. Despite certain limitations, the proposed method makes a significant contribution by pioneering a new direction for converting statistical data into tabular formats, laying the foundation for future advancements in this area.

The article is structured as follows. Section 2 is devoted to related works. Section 3 explains the proposed algorithm. The architecture of the modified GAN, which is considered an important part of the algorithm, the additional loss function used in it, and the formulas for calculating it are presented. Section 4 briefly provides information about the evaluation criteria and their calculation formulas. Section 5 presents the results of the evaluation based on the selected evaluation criteria to assess the effectiveness of the proposed method and compares them with the Gaussian Multivariate (GM), Synthetic Data Vault (SDV) and Conditional Tabular Generative Adversarial Network (CTGAN) methods. The reason for comparing with different methods, even though the proposed method has no analogues, is also explained at the end of Section 5. This chapter also provides a discussion of the results. Section 6 provides the conclusion of the article and outlines future work.

## 2. Related Works

The number of studies on synthetic tabular data generation has increased drastically in recent years [12]. Among them, methods such as CTGAN [13], Tabular Variational Auto encoders (TVAE) [14], Large Language Model-based [15] and Transformer-based hybrid methods [16] have been found to be the most effective. However, since these works are beyond the scope of this research work, the information in detail may be found in the literature [17,18,19,20].

The closest works to the proposed work are statistical and probabilistic methods. Therefore, the works conducted in this direction will be considered. Based on the statistical distributions and correlations of the variables in the real data, the statistical and probabilistic-based approaches create synthetic data. This type of approach is used by algorithms such as bootstrapping [21], the Bayesian models [22] and the probabilistic Bayesian networks [23]. These methods rely on their specific distribution functions to create synthetic databases. As a result, they are unable to generate tabular data directly from arbitrary statistical summaries of real datasets.

To our knowledge, only two methods that are close to ours have been developed so far [24,25,26]. Authors of [24] named their algorithm Synthea (Synthetic Health Data Generation). This is an open-source project designed to simulate realistic, synthetic patient health records for research, software testing and training purposes. Synthea uses a rule-based approach to model patient behavior, disease progression and healthcare encounters. It begins by generating a population of synthetic individuals. Demographic attributes such as age, gender and location are assigned based on real-world statistics to ensure realism. Environmental factors, including geography, access to healthcare and regional epidemiology, are configured based on external datasets. The algorithm uses disease modules that define the natural history of illnesses based on clinical guidelines and epidemiological data. Each disease is represented as a finite state machine with nodes and transitions. Nodes represent states (e.g., “Healthy”, “Mild Disease”, “Severe Disease”), and transitions are triggered by probabilities, time progression, or external factors. Disease progression and treatment are guided by evidence-based clinical guidelines, ensuring that simulated scenarios align with real-world healthcare practices. However, the quality and accuracy of the data generated by this method are highly dependent on the clinical guidelines, epidemiological data and expert knowledge used to build the state-transition models. Collecting and organizing these rules and knowledge requires the intervention of medical professionals. Also, knowledge preprocessing processes require a lot of time and effort. And this is the disadvantage of the method. Refs. [25,26] present a synthetic data-generation approach that uses statistical information of data and deep learning algorithms. This method involves modifying the primary database, which is initially developed based on statistical data, through the application of a specialized shuffle algorithm. The shuffle algorithm is designed to rearrange or manipulate the data in a way that ensures the resulting dataset meets predefined criteria for quality and realism, ultimately achieving a satisfactory outcome. Following the transformation process, the modified dataset is evaluated using a neural network. The neural network is utilized to assess the dataset’s quality, ensuring that it retains meaningful patterns, adheres to expected distributions, and maintains coherence with the original statistical characteristics. The evaluation result of the neural network is used as a loss function to adjust the parameters in the shuffling algorithm. However, one disadvantage of this strategy is the difficulty in reaching convergence, as the training loss function of this deep neural network depends on a shuffling technique, leading to long training times.

Thus, a new approach that uses statistical information and deep learning models was proposed to fill this research gap.

## 3. Proposed Methods

The original GAN is composed of two parts: a generator neural network and a discriminator neural network. The generator tries to trick the discriminator by producing samples from random noise, while the discriminator attempts to accurately differentiate generated and real data. In this way, these two networks compete in a zero-sum game, in which one part’s gain is proportional to another’s loss. The generator takes random noise as an input, and the discriminator receives generated and real data, respectively. The basic loss function is an adversarial or Minimax loss function. The terms min and max will stand for generator loss minimization and discriminator loss maximization, respectively. Expanding upon the basic model, we suggested three significant enhancements: (1) a modified generator network architecture with two inputs was designed, (2) a new loss function was used to train a generator neural network. Furthermore, to produce fully synthetic data, a unique training network method was proposed, which will be explained in Section 3.1.

### 3.1. Proposed Algorithm

**Figure 1:**
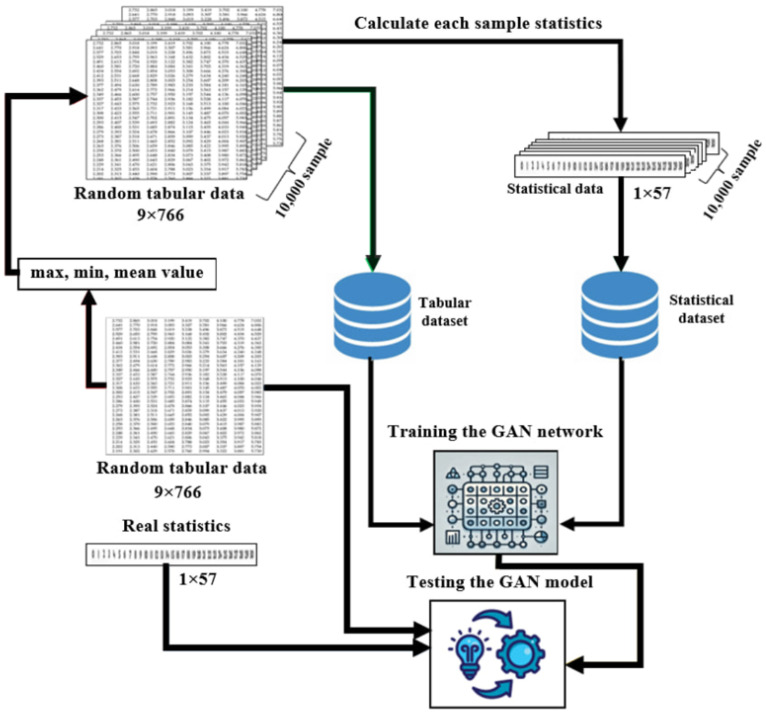
Steps of generating synthetic tabular data.

Before presenting the algorithm of the suggested method, the main idea of the work should be described in greater depth. First of all, the goal of the proposed work, unlike previous methods, is to generate synthetic data based solely on given statistical data without directly accessing the database. In contrast to conventional statistical or probabilistic methods, tabular data are generated using a Deep Neural Network (DNN) rather than complex mathematical/statistical formulas. In this method, DNN is trained on a dataset which consisted of statistical data and tabular data to convert statistical data into tabular data. In other words, this approach is similar to text-to-image (DALL-E, OpenAI) or voice-to-text techniques. The GAN first learns to generate a table based on statistical data, then based on the given table, it has the same statistical properties, but produces tabular data with slightly different row values. Therefore, the database needed to train this network consists of two parts, a database of tables and the corresponding statistical properties of these tables. Each step of this algorithm is described in Figure 1.

The first step is generating a dataset of statistical information. Since the tabular data are not given, first of all, random tabular data are generated. However, in order for the network to learn faster, the randomness of the data must be limited. This indicates that statistics that are similar or at least close to those contained in real datasets can be utilized to generate these raw data. For example, maximum, minimum and mean values of each column, percentage of elements with values greater than or less than a threshold value, etc., can be used to create raw tabular data. However, during this process, it should be noted that there will be at least one datum for each class.

More precisely, if a real dataset has y target classes, the outcome/target column of synthetic data must contain at least one element for each class. After generating random tabular data, all tabular data will be stored in separate files to compose the tabular database. Subsequently, the statistics of each table in the generated tabular dataset will be generated. All the values of the generated statistics will be combined into a single row and this row will be stored in a .csv file. As a proof of concept, the real dataset and its corresponding statistical information were taken. These real data and their statistics are taken away for testing the network performance, or as a ground truth.

The second step is to train the GAN with two datasets, i.e., tabular and statistical dataset. This network is a modified version of the GAN, which has two inputs. Furthermore, for training this GAN, an additional loss function for the generator network will be used. The details of the modified GAN and additional loss functions are given in Section 3.3 and Section 3.4, respectively.

The third step is the final step or evaluation step. For evaluation, different methods are used and their descriptions are provided in Section 4.

### 3.2. Modified GAN Architecture

A Generative Adversarial Network is made up of two parts, the generator and the discriminator, that are trained together via adversarial training. The discriminator is used to improve its ability to discern between actual and fake data, and the generator takes random noise as input and generates synthetic data close to the real data. Moreover, it attempts to generate data during training that the discriminator is unable to distinguish from real data. In the simplest terms, the two networks are playing a game against one another: the generator wants to generate believable fake data, while the discriminator is trying to differentiate between real and fake data. Over time, the generator produces ever-better data as a result of this adversarial process.

**Figure 2:**
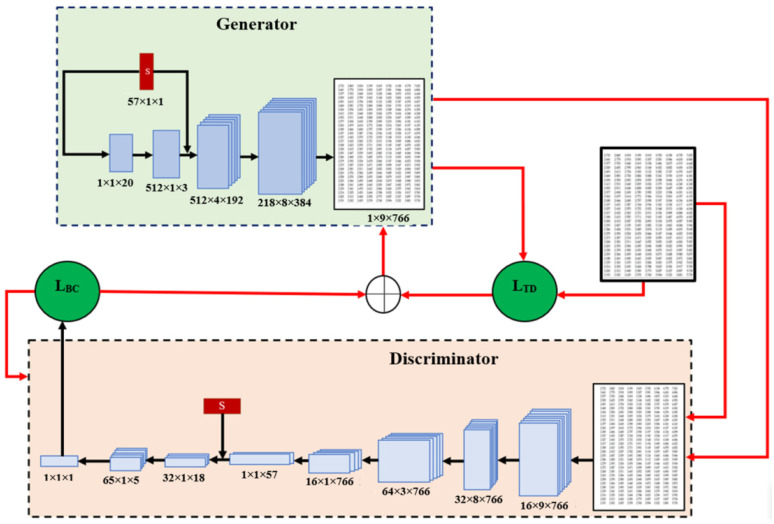
Architecture of modified GAN.

Random noise will constitute the network’s input, and by adding noise and sampling from various locations within the target distribution, GAN can produce a variety of data. However, our goal is to produce tabular data featuring all the internal relationships that are precisely like the provided statistical data; in other words, the generator should produce such tabular data that, even if its value differs from real data, the statistical properties should be the same as the given statistical data. In order to achieve this, statistical data were added to the generator’s input in addition to random noise as illustrated in Figure 2. It can be seen from the Figure 2 that statistical data are first fed into the generator’s input, and after several layers statistical data are fed again. The purpose of inputting twice is to focus the network on the information being entered. This should be done as far as possible after the first layers, because in the subsequent layers the network begins to recover the properties of the table. However, for input after the first layer, the output of the first layer and the statistical data form must be suitable for concatenation. However, this requires the use of very large filters in the first layer, which dramatically increases the number of parameters of the network, so it is advisable to input after two layers instead of one. Concatenated data are then used to produce fake tabular data. Generated and corresponding real data are sequentially entered into the discriminator. Then, the discriminator begins to identify features of both data. Appropriate statistics are entered into the discriminator when reaching a certain nth layer. In this instance, the statistical data and feature map of the last layer are concatenated and transmitted to the next layer. At the end of the training, the discriminator comes to a conclusion about how different the fake data are from the real ones. This conclusion is presented in the form of a loss function (2). The corresponding loss function (1) is sent to the discriminator and generator, respectively, to back-propagate.

(1) $$\nabla_{\theta_{d}}\frac{1}{m}\;\sum_{i=1}^{m}\left[logD\left(x^{\left(i\right)}\right)+log\left(1-D\left(G(z^{i})\right)\right)\right]\;\;\;\;\;\;\;\;\;\;\;\;\;\;$$

Moreover, the loss function of the generator is presented below (2):

(2) $$\nabla_{\theta_{d}}\frac{1}{m}\;\sum_{i=1}^{m}log(1-D\left(G(z^{i})\right))+L_{TS}$$

Here, $L_{TS}$ is the custom loss function; we named it the Table similarity (TS) loss function. A comprehensive description of the custom loss function is provided in Section 3.3.

### 3.3. Custom Loss Function

In conventional GAN Binary Cross Entropy (BCE), a loss function is used. The purpose of using the BCE loss function for the generator is that the discriminator should evaluate the probability of real-likeness of the images generated by the generator. However, in this particular case, not only the probability of real-likeness but also resemblance to the real table should be estimated. It is not required to generate a completely new table that combines the attributes of multiple tables, but rather a table that has precisely the same statistical properties and correlational relationship as the appropriate table. In other word, the purpose of the model is converting statistical data into tabular data.

The output shape of the generator is 9 × 766 in our case. In contrast to previous table-generating GAN approaches, the generator in this network produces a sample table that includes data of multiple patients. In our example, 766 patient data are generated all at once.

Although it looks like a 9 × 766 pixel image, mean squared error (MSE) and mean absolute error (MAE) losses could not be used to evaluate resemblance of real and synthetic samples. The images are compared pixel by pixel; the MSE or MAE loss function is typically employed to assess the similarity of the images. Nevertheless, unlike images, tables should be similar row-wise, not pixel-wise. This is due to the fact that the statistical information of the table remains unchanged even if the rows’ positions in the table alter. For this reason, the most optimal way to evaluate the similarity of tables is to compare them row by row.

Tabular data are typically compared using the Kullberg–Leibler (KL) loss function, the Jaccard Index and the cosine embedding loss function. However, employing these functions comes with a variety of issues. Specifically, because the Jaccard index relies on sets, it cannot be utilized directly as a loss function. The loss function for cosine embedding is designed for binary data and is not applicable to non-binary data. The KL loss function is a relatively appropriate loss function. However, the network has trouble converging and is challenging to train when KL is utilized as a loss function. Due to this, we proposed a new loss function to assess the similarity of tables, and we named the new loss function TS loss function. The new loss function is easy to calculate and allows you to compare table rows even when they change their positions. The sequence of its calculation is as follows:

First step—computing the sum for each row: Let the real table be represented as a matrix *R* and the synthetic table be represented as a matrix S. In addition, where *x_nm_* is the element in the *n-th* row and *m-th* column of *R*, *y_nm_* is the element in the *i-th* row and *j-th* column of *S*. In our case, the matrices have 766 rows and 9 columns.

The sum of *R* matrix elements in the *n-th* row is given by:

(3) $$r_{n}=\sum_{m}^{9}x_{nm}$$

The sum of S matrix elements in the *i-th* row is given by:

(4) $$s_{i}=\sum_{j}^{9}y_{ij}$$

The above summation for each row *n* and *i* will be performed, where *n* = 1, 2, …, 9 and *i* = 1, 2, …, 9. Resulting in two rows of sums, *r* and *s*.

Second step—computing the sum for each column: The sum of *R* matrix elements in the *m-th* column is given by:

(5) $$R_{m}=\sum_{n}^{766}x_{nm}$$

For synthetic data, the sum of elements in the *j-th* column is given by:

(6) $$S_{j}=\sum_{i}^{766}y_{ij}$$

The above summation for each column *m* and *j* will be performed, where *m* = 1, 2, …, 766 and we will receive two rows of sums, R and *S*.

Third step—defining the mean of vertical subtractions: After computing the sum of columns of each datum, element-wise subtraction will be performed. As a result, the set of subtractions is created, such that *C* = {$\left|{\;R}_{m}-S_{j}\right|$}. Next, the mean value μ of the subtractions is calculated:

(7) $$\mu =\frac{1}{9}\sum_{k=1}^{9}C_{k}$$

where $C_{k}\;$ represents the *k-th* value in the set *C*.

Fourth step—calculating minimum subtraction for each row: The module of subtraction of each $r_{n}$ element from each $s_{i}$ is calculated: $\left|r_{n}-s_{i}\right|$. Then for each *n-th* element of *r*, the minimum value of the subtractions module will be found:

(8) $$\delta_{n}=\underset{i}{\min}\left(\left|r_{n}-s_{i}\right|\right)$$

Thus, a set M which consists of minimum values $\delta_{n}$ will be formed: $\;M=\{\delta_{1},\delta_{2},\ldots ,\delta_{n}\}$, where, *n* = 1, 2, …, 766.

Fifth step—finding percent of repeated minimum value: Now, 766 minimum values are obtained, of which *N* has equal value. Then, the repeated minimum value percent is equal to P=N/766.

Sixth step—defining the mean of minimum values: From the *M* set, its mean value will be defined:

(9) $$\nu =\frac{1}{766}\sum_{n=1}^{766}\delta_{n}$$

Seventh step—calculating horizontal loss: Finally, horizontal loss will be found:

(10) $$D=\frac{\nu }{P}$$

Overall loss for one sample will be defined as:

(11) $$L_{onesample}=D+\mu$$

During training *L*_1,*onesample*_, *L*_2,*onesample*_ and *L*_*k*,*onesample*_ loss values are obtained for each instance in a batch, where *k* is the number of instances in the batch. The final step is to take the square root of the mean value of the loss function for the batch:

(12) $$L_{TS}=\sqrt{\frac{1}{k}\sum_{l=1}^{k}L_{l}}$$

### 3.4. Dataset

The Pima Diabetes Database (PDD) was chosen to use as a dataset in our experiment. The dataset is named after the Pima Indians, a Native American group from the Pima Country region in Arizona, USA. It was collected as part of a study conducted by the National Institute of Diabetes and Digestive and Kidney Diseases (NIDDK) to investigate diabetes prevalence and related factors among this population [27]. The dataset contains 766 samples, with each instance corresponding to a patient record. Each record is described by 8 features, including pregnancies, glucose, blood pressure, skin thickness, insulin, BMI, diabetes pedigree function, age and the binary outcome variable.

The proposed method does not necessitate the use of an actual database; instead, it relies on statistical information about the database. However, for the purpose of evaluating the generated database, a real database is used. More precisely, its statistical information was extracted and used to train the network. A tabular database is employed to test the network and for comparative evaluation. The primary reason for selecting this database was that the model is designed to generate a small-scale database.

From this database, the maximum and minimum values, percentage of risky values and OR value for each column were determined. A total of 57 values were extracted. A dataset of 10,000 statistics that differed by 1% from that statistics was then generated. For this, the random generator from the Python library was used.

As a result of this, the dataset consisting of 10,000 data, each with 766 rows and 9 columns, was generated. These dataset was almost, similar to the PIMA dataset, or rather, the statistics differ by 1% and this dataset is then called the primary dataset.

## 4. Evaluation Criteria

Once the synthetic data are developed, assessing their quality is the most crucial stage. Even though a universal and standardized assessment methodology for synthetic data quality evaluation has not yet been established, several evaluation metrics and methodologies have been proposed and used [28,29]. Based on their intended purpose, the evaluation techniques used to evaluate the quality of the data can be categorized into three classes:

1. Fidelity-assessment metrics.
2. Utility-assessment metrics.
3. Privacy-assessment metrics.

However, two metrics have been utilized in this work, and the reason of selection is provided in the “Results and Discussion” section.

### 4.1. Fidelity-Assessment Metrics

There are numerous metrics proposed and used to evaluate fidelity [29], and all of them are categorized into three groups: statistical test for numerical attributes, statistical test for categorical attributes and distance calculation. Every category includes many metrics. One of the most significant and commonly used method is considered in this work. More precisely,

1. For statistical test for numerical attributes: Student’s *t*-test.
2. For statistical test for categorical attributes: Chi-square (χ^2^) test.
3. For distance calculation: Jensen–Shannon divergence.

#### 4.1.1. Student’s *t*-Test for the Comparison of Means

The formula for Student’s *t*-test is defined as (13):

(13) $$t=\frac{{\bar{x}}_{1}-{\bar{x}}_{2}}{\sqrt{\left(\frac{\sigma_{1}^{2}}{n_{1}}+\frac{\sigma_{2}^{2}}{n_{2}}\right)}}$$

where *t* = Student’s *t*-test, *x*_1_ = mean of first group, *x*_2_ = mean of second group, σ_1_ = standard deviation of group 1, σ_2_ = standard deviation of group 1, *n*_1_ = number of observations in real dataset, *n*_2_ = number of observations in synthetic dataset.

In cases of multiple-column tabular data, a separate *t*-test should be carried out for every column. In ref. [30], it is mentioned that the critical value can be chosen as 0.05. This means that if the test’s *p*-value is greater than this amount, it can be considered as a mean of real data and the synthetic features are similar. Otherwise, the attributes of the two datasets will be different from each other.

#### 4.1.2. Chi-Square Test

When comparing the independence of categorical features, Chi-square analysis is employed. If the synthetic data attributes maintain the characteristics of the real data attributes, the null hypothesis is accepted and for this the *p*-value was set as 0.05. The formula of the Chi-square test is given below:

(14) $${ℵ}^{2}=\sum \frac{{\left(S-R\right)}^{2}}{R}$$

where *S* is statistical data and *R* is real data.

#### 4.1.3. Wasserstein Distance

The Wasserstein distance estimates the distance, in a given metric space, between two probability distributions. The *p-th* Wasserstein distance is given by:

(15) $$Wass\_dist(r,s)=\int_{-\infty }^{+\infty }\left|R-S\right|$$

*R* and *S* are the cumulative distribution functions of the real and synthetic datasets, respectively.

#### 4.1.4. Univariate Resemblance Assessment (URA)

In ref. [29], the authors introduced a verbal evaluation method of the generative model performance. Firstly, statistical tests and distances are calculated for every feature of the dataset, and then the threshold value is used. It is determined whether each character meets the h0 hypothesis or not. After that, the number of features that fulfill the requirements is counted. The generative model is labelled “Excellent” if this number is higher than 50% percent of the number of all the features. If less than 50% percent, it is labelled “Good”; if the number is zero, then the model is labelled “Poor”. After labelling the model based on each metric, a URA score is calculated. For this purpose, labels are converted to numerical values as follows: “Excellent”  =  3, “Good”  =  2, “Poor”  =  1. And next, every number is multiplied by a proportionality coefficient.

The proportionality coefficient is defined according to this formula:

(16) $$M=\frac{1}{n}$$

where *n* is the number of metrics used to evaluate fidelity.

In our case *n* = 3, so the converted numbers will be multiplied by 0.33 and thesum of all multiplications will be calculated. The outcomes are first rounded, and the resulting numerical value is subsequently determined. This numerical value is then translated into descriptive categories as follows: “Poor” for a value of 1, “Good” for a value of 2 and “Excellent” for a value of 3.

### 4.2. Utility-Assessment Metrics

Usually, the synthetic dataset is mainly used to train machine learning or deep learning algorithms. For this reason, Train on Synthetic, Test on Real (TSTR), Train on Real, Test on Real (TRTR) and Train on Real, Test on Synthetic (TRTS) methods are used to evaluate the usefulness of synthetic data. These methods are described in detail in the following:

The TSTR metric involves training a machine-learning model on synthetic data and then testing its performance on real data [31]. TSTR is a more important metric than TRTS because the purpose of generating synthetic data is using them for training AI algorithms. For this reason, the TRTS method was not used to evaluate utility.

Obviously, the accuracy of the model depends not only the dataset, but also on the Machine Learning (ML) model. This means that valuating synthetic data utility only with one ML model is insufficient. For this reason, several ML models were used to assess the utility metric, namely, Random Forest (RF) classifier (1), k-Nearest Neighbors (KNN) (2), Decision Tree (DT) classifier (3) and Multilayer Perceptron (MLP) classifier (4). The parameters for these models were configured as follows:

1. RF: Number of estimators = 88, Maximum depth = 11, Random state = 28 and Criterion = “Gini”. All other parameters were set to their defaults.
2. KNN: Number of neighbors = 4, Metric = cosine value. All other parameters were set to their defaults.
3. DT: Maximum depth = 7, Random state = 27, Maximum features = 2. All other parameters were set to their defaults.
4. MLP: Hidden layer sizes = (32,128,256), Maximum iteration = 30, Random state = 9, Verbose = 1. All other parameters were set to their defaults. The training process stopped if training loss did not improve more than 0.0001 for 10 consecutive epochs.

After training ML models, in order to evaluate model-classification performance traditional metrics, accuracy, precision, recall and F1-score were used.

### 4.3. Comparison with Other Methods

To assess the quality of the dataset generated by the proposed method, a comparative analysis was conducted. For this purpose, three synthetic database-generation methods are selected on the basis of their effectiveness. These methods include:

GM—a technique based on modeling the joint probability distribution of the data using multivariate Gaussian distributions.

SDV—a framework that leverages probabilistic modeling and statistical techniques to generate synthetic datasets while preserving the original data’s structural and statistical properties.

CTGAN—a generative model that employs GANs tailored for tabular data, enabling the generation of realistic datasets even in the presence of imbalanced and categorical data.

## 5. Results and Discussion

The training of the GAN is performed using the Adam optimizer. To mitigate the risk of mode collapse, the learning rate for the discriminator was set to 0.00005, while the learning rate for the generator was set to 0.0001. The batch size was fixed at 64. As outlined previously, two distinct datasets—statistical and tabular databases—were employed for training, each comprising 10,000 samples.

Throughout the training process, the GAN’s performance was continuously monitored to evaluate the network’s progress, and scatterplot graphs were generated to illustrate the correlation between two specific features (the fifth and seventh) from the tables produced in each batch. These scatterplots were then visually compared against those generated from the real datasets to assess the similarity between the real and synthetic data.

**Figure 3:**
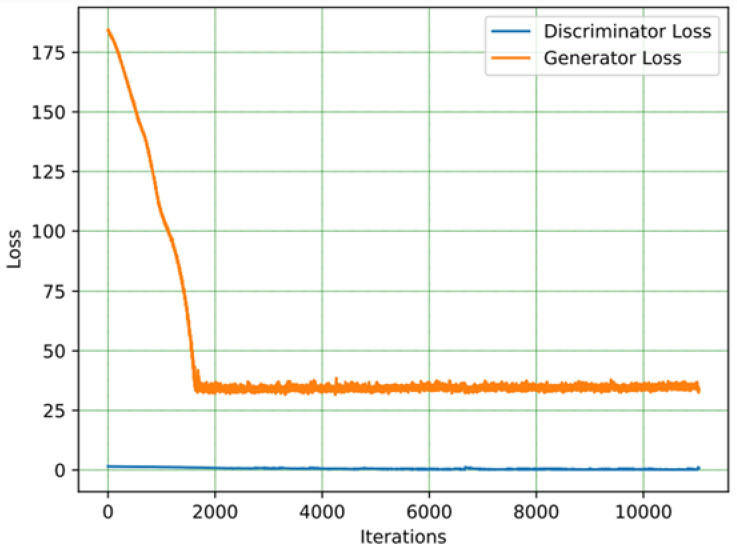
Results of generator and discriminator loss during training.

The network demonstrated improvements in performance up until the 70th epoch (10,920th iteration) (Figure 3). Beyond this point, the quality of the generated data began to decline; even Loss values remained the same. This prompted the decision to terminate the training at the 70th epoch to prevent overfitting and ensure optimal model performance.

After the training process, the next and final step was testing the performance of the generator. As a ground truth, the real PIMA diabetes dataset is used. Three criteria were used to evaluate the performance—fidelity, utility and privacy. The outcomes of these assessment techniques were as follows.

For fidelity evaluation of synthetic data, Student’s *t*-test, Chi-square and Jensen–Shannon distance estimation methods were utilized for every attribute of data.

In Table 1, the *p*-values derived from the statistical *t*-tests for each column are given. From the table, it can be seen that all features generated by SDV have *p*-values of Student’s *t*-test greater than 0.05, indicating similarities in mean values as real dataset features. *p*-values for 7 out of 8 features are higher than the threshold value; that is, GM also generated data with mean values very close to the real dataset; seven of the eight features generated by GM had *p*-values of *t*-test higher than the threshold value, which shows that they also have mean values close to the real data features’ means. In the data generated by the proposed method and CTGAN, only two features satisfied the required condition. The *t*-test showed that only a few features’ mean values were simulated closely using the proposed method and CTGAN. However, it is indicated in Section 4 that if less than half of the features’ *p*-values match the condition, the result of the test can be classified as “Good”; therefore, we rate the performance of the proposed and CTGAN methods as “Good” and the performance of the GM and SDV as “Excellent”.

The *p*-values acquired for every category attribute are displayed in the Chi-square test results. It can be seen from Table 2 that for all methods *p*-values were above the established critical level (>0.05), showing the acceptance of the alternative hypothesis (*h*1) and a statistical relationship between the two features. The performance was labelled “Excellent”, since the alternative hypothesis was accepted for every categorical attribute produced by every technique. The last point that should be noted is that our proposed method earned the highest score.

Table 3 shows the proposed and CTGAN techniques which produced data with Wasserstein distances less than 0.1 for every feature. Only one feature of the GM generated dataset and two features of the SDV generated dataset had a Wasserstein distance bigger than the threshold value. As previously stated, even though not all features fulfill the conditions set forth concerning closeness, it is enough that more than half of the features meet the criteria in order to classify the data as “Excellent”. For this reason, all approaches were categorized as “Excellent” for Wasserstein distances.

In Table 4, labels are provided for each fidelity metric, along with the overall URA performance. With the exception of CTGAN and the proposed method, every technique preserved the univariate characteristics of real data accurately. Even the proposed approach exhibits a worse performance, with Numerical Statistical Test total URA values similar to GM and SDV models. This means that using the proposed method, it is possible to create data whose statistical properties are very similar and close to those of real data.

For the utility-evaluation TSTR test, RF, KNN, DT and MLP models are used. For every model, accuracy, precision, recall and F1 scores were calculated. Table 5 and Table 6 present the information about the TSTR and TRTR test results.

**Figure 4:**
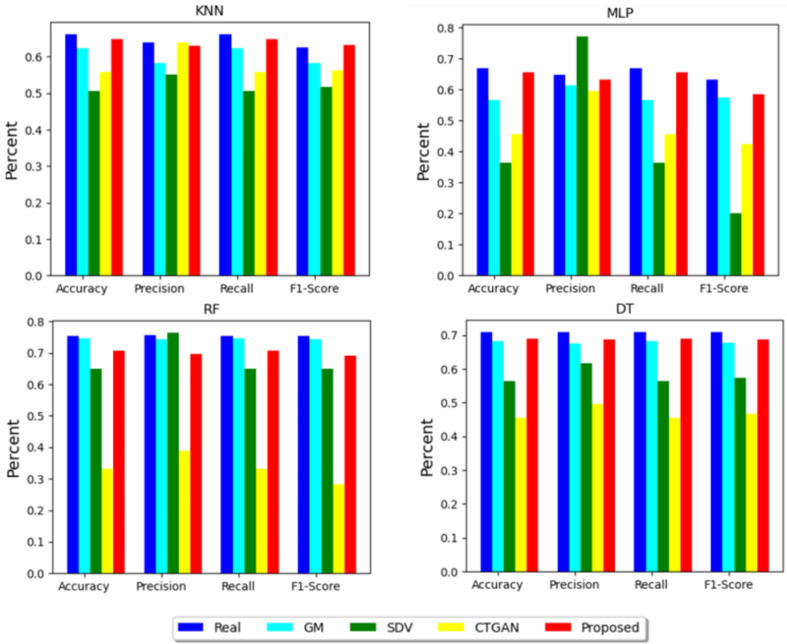
Results of TSTR test carried out with different ML algorithms.

The first row of the table shows TRTR results for every ML algorithm. From Table 5 and Table 6, it can be seen that, when trained using the dataset produced by the proposed method, three algorithms, namely KNN, DT and MLP, achieved the maximum performance. Thus, the proposed method gained the highest TSTR value in 3 out of 4 tests. Only in one case, i.e., RF, GM took first place in term of accuracy, recall and F1-score. However, the proposed method also performed well when TSTR was tested with the RF model, placing second among all models behind GM. The CTGAN model, whose architecture is closest to the proposed method, showed the lowest result in all cases. To analyze results visually, all results are plotted in the form of a bar graph (Figure 4). Concerning the variances in accuracy, precision, recall and F1-score values between the TRTR, it can be seen from the pictures that almost all values of the dataset created by the proposed method were the lowest when checked by DT, KNN and MLP methods. However, only when testing with the KNN model, the difference in precision values between TRTR and TST were marginally smaller than that of the proposed technique for CTGAN. Furthermore, the GM approach produced the smallest values for all parameters only in cases of the RF method test. Nevertheless, even when the dataset generated by the proposed method was tested by the RF method, the difference of all metrics of TSTR and TRTR was less than 0.1, which is a very acceptable outcome.

The Figure 4 shows that the approach is categorized as the “Excellent” if the difference in the test performance between TSTR and TRTR is less than 0.4. In our instance, the differences between all models (CTGAN excluded) on all metrics were less than 0.4 across all tests (KNN, DT, RF and MLP). CTGAN also performed well in 3 out of 4 cases, but obtained poor results when tested by the RF method. Therefore, the utility metrics of all methods were classified as “Excellent” for all approaches.

From the above results, the proposed method demonstrates promising results in terms of both similarity and usefulness. While it performs on par with the CTGAN algorithm in terms of similarity and generates a synthetic table satisfactorily similar to the real one under the URA criterion, it falls slightly behind the GM and SDV methods. However, its ability to outperform other methods in three out of four usefulness tests highlights its strength in accurately representing relationships between variables. Unlike GM, which can produce negative values that disrupt variable relationships, the proposed method maintains consistency, contributing to its superior usefulness scores. GM also showed results close to the proposed method, but usually there are negative values in the table generated using GM, and if one filters them using the thresholding method, they can still give values close to real, but they affect the relationships between variables. Therefore, the GM method showed a slightly lower result.

Since the proposed method does not directly use a real table to generate the synthetic table, this method is not evaluated according to the security criteria. Because it does not rely on real tables or personal data, ensuring that no actual patient information is stored or processed. So, from a security perspective, the method has significant advantages, this makes it a robust choice for privacy-preserving synthetic data generation.

This proposed method was the first step towards opening up an area with great potential for the future. By enabling the generation of realistic tabular datasets from statistical data, these algorithms bridge a crucial gap, providing accessible, privacy-compliant and cost-effective alternatives to real-world datasets across diverse applications. The availability of extensive medical, economic and financial databases for training machine learning algorithms has the potential to significantly accelerate advancements in research within these fields, fostering unprecedented progress and innovation. However, much work needs to be conducted in the future for this area to develop. The proposed method showed the better results in terms of fidelity and usefulness as compared to other existing methods. In particular, the network is trained only to recognize specific statistical data and convert them into a table. Each time different types of statistical data are used, one has to train the network again. Furthermore, as the number of variables in the database increases, training the network also becomes more difficult. The network architecture needs to be changed to make it relatively easier to train. The method proposed in this article is designed only for creating a small database; the same method can be adapted for a large database, and in our future work we aim to consider the issues of creating a large database.

## 6. Conclusions and Future Work

This article introduced a novel approach for generating synthetic tabular datasets derived from statistical data. The proposed method is evaluated based on fidelity and utility characteristics. While not all attributes of the synthetic table met the fidelity requirements for replicating the real table, the overall similarity is rated as good. In terms of utility, the method excelled, achieving good results in the KNN, DT and MLP tests. The TSTR accuracy differed minimally from that of the real database, with deviations of only 0.12%, 0.19% and 0.13% for KNN, DT and MLP, respectively. These utility scores surpassed those of all three compared models, highlighting the proposed method as a superior choice for training machine learning models.

Despite the aforementioned advantages, several improvements can be implemented in future work: enhancement of network architecture, adaptation of the algorithm to generate large-scale databases and optimization of network parameters to facilitate efficient training, even as the number of variables in the table increases.

## Figures and Tables

**Table 1:** Student’s *t*-test results.

Attributes\Model Names	Proposed Model	GM	SDV	CTGAN
Pregnancies	0.0000	0.0271	**0.4539**	0.0000
Glucose	0.0000	**0.9834**	**0.6115**	0.0000
Blood Pressure	0.0000	**0.3075**	**0.8030**	**0.1696**
Skin Thickness	**0.1127**	**0.8747**	**0.9245**	**0.6030**
Insulin	0.0000	**0.8664**	**0.6294**	0.0000
BMI	0.0019	**0.7159**	**0.8157**	0.0000
Diabetes Pedigree Function	0.0009	**0.8929**	**0.1311**	0.0000
Age	**0.3811**	**0.3766**	**0.6342**	0.0010

**Table 2:** Chi-square test results.

Model Names	Proposed Model	GM	SDV	CTGAN
**Outcome**	**0.977744**	0.9486	0.8987	0.901

**Table 3:** Wasserstein distance values.

Attributes\Model Names	Proposed Model	GM	SDV	CTGAN
Pregnancies	**0.077049**	**0.050503**	0.210039	**0.090343**
Glucose	0.139552	**0.029401**	**0.022376**	0.211049
Blood Pressure	0.119209	**0.077486**	**0.054027**	0.135793
Skin Thickness	0.166620	**0.082580**	0.192674	**0.063013**
Insulin	**0.047145**	0.381628	0.444365	0.161786
BMI	0.155688	**0.026149**	**0.053898**	0.111652
Diabetes Pedigree Function	0.176867	**0.051625**	0.125081	**0.057591**
Age	**0.086909**	**0.022935**	0.385533	**0.031314**

**Table 4:** Fidelity assessment’s overall results.

Approach	Numerical Statistical Tests	Categorical Statistical Tests	Distance Calculation	Total URA
Proposed	Good	Excellent	Good	Good
GM	Excellent	Excellent	Excellent	Excellent
SDV	Excellent	Excellent	Excellent	Excellent
CTGAN	Good	Excellent	Excellent	Excellent

**Table 5:** Utility assessment with KNN and DT.

Method Name	KNN				DT			
	Acc. (%)	Prec. (%)	Recall (%)	F1 (%)	Acc. (%)	Prec. (%)	Recall (%)	F1 (%)
Real	0.6623	0.6388	0.6623	0.6256	0.7078	0.709	0.7078	0.7084
GM	0.6234	0.5839	0.6234	0.5824	0.6818	0.6739	0.6818	0.6764
SDV	0.5065	0.5511	0.5065	0.5167	0.5649	0.6167	0.5649	0.5735
CTGAN	0.5584	**0.6384**	0.5584	0.5627	0.4545	0.4954	0.4545	0.4659
Proposed model	**0.6494**	0.6304	**0.6494**	**0.6326**	**0.6883**	**0.6859**	**0.6883**	**0.6873**

**Table 6:** Utility assessment with RF and MLP.

Method Name	RF				MLP			
	Acc. (%)	Prec. (%)	Recall (%)	F1 (%)	Acc. (%)	Prec. (%)	Recall (%)	F1 (%)
Real	0.7532	0.7554	0.7532	0.7542	0.6688	0.648	0.6688	0.6307
GM	**0.7468**	0.7426	**0.7468**	**0.7438**	0.5649	0.6122	0.5649	0.5738
SDV	0.6494	**0.7648**	0.6494	0.6494	0.3636	0.7712	0.3636	0.2017
CTGAN	0.3312	0.388	0.3312	0.2812	0.4545	0.5942	0.4545	0.4246
Proposed	0.7078	0.6966	0.7078	0.6926	**0.6558**	**0.6311**	**0.6558**	**0.5831**

## Data Availability

The original contributions presented in the study are included in the article; further inquiries can be directed to the corresponding authors.
